# The Profession, the Public, and the Politicians

**Published:** 1917-07-21

**Authors:** 


### The Hospital

The Workers' Newspaper of Administrative Medicine and Institutional Life, Administration, National Insurance and Health.

No. 1624, Vol. LXII. SATURDAY, JULY 21, 1917. PRICE ONE PENNY

### THE PROFESSION, THE PUBLIC, AND THE POLITICIANS.

The nation just now is hardly in a happy frame of mind. From high quarters the exhortation comes to us to be "steady," but our governing authorities do not better the instruction by setting us a good example. Questions of general politics do not concern us here except in so far as they touch the interests of the medical profession and its related constituencies. Some of these interests, however, are distinctly engaged in questions now widely canvassed, and we can hardly avoid a frank discussion of them. If such a discussion involves references to wider political issues this is in the nature of things, and no obligation of silence arises from it. What chiefly affects every one of us is the winning of the war with all possible expedition, and, as contributing to this, the removal of hindrances to dispatch. Such considerations make the fate of parties and of individual politicians a matter of little concern. The motto for the moment is surely found in the words of Burke as quoted by Mr. Asquith in his recent speech in the House of Commons, "Let us pass on. For God's sake let us pass on." We have some special warrant for emphasising how inconsistent with such a mental decision is a digging up of the past and a scrutiny of its failures in the light of fuller knowledge. If we are satisfied that it is impossible to combine the two aims the choice leaves no room for hesitation. The election must be made for the main issue even though it involve some loss and disadvantage on the lesser claim.

There is another demand having for its basis a firmly grounded experience, and specially acute in existing circumstances. It is that discussions take an end. Truth and justice for individuals let us have by all means, so far as these are possible in human affairs. But the settlement must come with due heed to claims which have a still wider scope, and must be reached with reference to the pressure of current events. Even in the smooth days of peace this doctrine is one of common sense. How much more urgent is it in the imperative circumstances of war! When the need for action is acute, open discussions, reiterated inquiries, and recalled decisions are paralysing agencies, and they have a wide play outside the field of their immediate application. Our enemies have been largely credited with an inability to appreciate the mental qualities of other nations, and to have committed in consequence serious errors of policy. In the light of some recent events it may fairly be asked whether our own governing authorities have a very apt measure of the mentality of the people whose affairs they are charged to direct. The time is surely one for the facing of facts, for strong and clear decisions, for the leaving of things which are behind, and for the closing of questions which in the circumstances of the moment can carry no practical or fruitful values. "Let us pass on. For God's sake let us pass on."

One of the most unhappy distractions of the moment is certainly the Mesopotamia affair. It has been investigated by a tribunal appointed for the purpose, and the tribunal has delivered its decisions. For ourselves, we accepted the judgment as one framed by competent men having access to the best sources of information, and, unless we are much mistaken, public opinion adopted a similar view. Yet now we are told that the "legal rules of evidence" were not strictly observed, that individuals have been "most unjustly attacked by the Commission," and that the method adopted "was not the proper method for dealing with these great affairs of State." Consequently the whole question is once more to be cast into the melting-pot, a new tribunal is to be set to work with a further period of distraction and uncertainty, and possibly, at least in some instances, yet a third inquiry by courts-martial or by Cabinet Ministers is to emerge. We wish to state an emphatic judgment that this announced procedure will exercise a most prejudicial influence on the mind and will of the nation. Anxious as we are to avoid injustice to individuals, we are bound to say that the time is not one for technical refinements and subtle qualifications, and that the practice of these expedients is more likely to produce exasperation than satisfaction. Further, at least as we think, such methods are entirely unnecessary and out of place. The opportunity for decision and judgment has arrived, and the mental atmosphere of the nation would be cleared by its exercise. If those charged with this duty hesitate to meet it, the time has come for more resolute counsellors. We repeat the conviction that these hesitations and delays and repeated debates cannot be reconciled with exhortations to "steadiness." The practice overshadows and neutralises the teaching.

What is the position of the military medical officers immediately concerned? They have openly been censured by the Commission for failures which have carried serious consequences. Whatever may be said in justification or excuse can be said to those set in authority over them. Further, these latter are in a position to know how far service in other directions may reasonably be set against present failure, and to judge what is the value of each officer to the country in the present crisis. Is it not the business of the Secretary of State for War to review all these considerations, as they can be set before him by the appropriate officials, and to close the debate? What is needed is to get rid of men who have proved themselves incompetent, and not to cashier good servants who have made a single error of judgment in difficult circumstances. Let the decision be swift and stern both in the one direction and the other. To manufacture "criminals" and to howl for "scapegoats" is a proceeding natural to the sensational press. But if Governments are "steady" themselves they may count on "steadiness" in the British public, which has more confidence in resolution and decision than in a capacity for escaping responsibility.

